# Foreign Correspondence

**Published:** 1872-05-15

**Authors:** 


### Foreign Correspondence.

VIENNA, MAY 1st, 1872.

The general plan and arrangement of that vast chain of buildings which together form the Vienna Allgemeinen Kranken house, or General Hospital, has been so often described in our American journals that it must be quite as familiar to those of the profession who have not been here as to those, more fortunate, who have been able to avail themselves of the extensive field for clinical study and observation which its wards afford.

The hospital buildings, low, old-fashioned structures, although very well arranged and adapted for the purpose, present nothing either striking or attractive in external appearance. The handsome and extensive gardens which occupy the central space enclosed within the circuit of buildings, forms the most characteristic and pleasing feature of the institution. The cool shady walks—the smooth green lawns, and the tastefully arranged flower beds, remind one of some handsome private park or pleasure grounds, rather than of a hospital enclosure. A second glance, however, at the occupants of the numerous seats and benches which line the walks, recalls one at once to the true nature of their surroundings. The convalescent patients, as soon as they are able to either walk, crawl, hobble, or be carried out, gather here in the gardens to enjoy the revivifying influence of the fresh air and sunshine—their odd and rather ghostly-looking hospital attire of white cotton cloth wound loosely about the person, forming a strange contrast to their out-door surroundings. Even the otherwise bare and rather gloomy hospital wards derive a certain bright, cheerful aspect from the gardens into which they look.

These grounds present a busy scene of a morning, as the students come gathering in from all directions like a vast army to a muster. Breaking up into little knots and groups, however, they separate again, and, into so many different destinations do they find their way, that, with perhaps two or three exceptions, in no one clinic would we find more than fifteen, twenty, or possibly twenty-five students.

The courses of instruction for the Summer Semester, which began on the 15th of April, are now fully under way. The general clinics and lectures of Profs. Hebra and Billroth, Braun, Arlt, etc., draw their usual crowds of listeners, while the multitudes of private courses offer their usual facilities for special study and investigation.

The demand created by the large number of transient visitors and students of all nations who are constantly arriving here, with perhaps but a month, six weeks, or two months to remain, and who are consequently willing to pay almost any prices in order to obtain access to the particular departments in which they are especially interested, enables almost any of the interns or assistants who may have a few wards under their charge, to attract a class of students. Most of these gentlemen undoubtedly endeavor to make their classes as interesting and instructive to their patrons as possible. Unfortunately, however, many of them, although otherwise well fitted for the positions which they occupy, are entirely lacking in any natural tact or ability for teaching.

Not a few of these gentlemen, too, seem entirely to forget the important fact that it is not so much *their individual* reputation and acquirements which draw the students, as the valuable material which is only to be reached through them. There are quite a number of these private courses, also, conducted on the evident principle of first squeezing all the money possible out of their victims, and then giving them just as little instruction in return therefor as possible, with the idea, apparently, of thus obliging them to take another course in order to complete the unfinished subject—very much on the same principle that a gambler, after having played once and lost, must try again in order to recover his loss, and then having lost a second time, he cannot, of course, give up without one more effort to win it all back.

I might mention here a single case in illustration. A certain young professor, the son of one of the best known, leading members of the Vienna faculty, gives a private course of twelve lessons, one hour each, on auscultation and percussion, for which he charges the moderate sum of fifty guilders, or twenty-five dollars. One of his late victims, on venturing to mildly remonstrate with him on the rather meager and unsatisfactory character of the instruction received, and the very sparse amount of material offered for practice and illustration, received the suggestive reply that in his next course he should take the class into another ward, where there was a much better supply of material, and that he should, therefore, be able to make it much more profitable and interesting. My friend *didn't bite*, however. Very few do, I think, a second time. A few words of advice and assistance from any of the students who have been here for a short time will, however, readily enable the stranger to avoid these *sharks*.

Unfortunately, however, the places in all the best and more favorite courses in all departments, as Schrotter on throat and chest affections, Politzer on the ear, Jager and Arlt on the eye, etc., are all engaged usually for several months in advance. A little time and patience is therefore required in getting started, unless, indeed, the precaution has been taken to write on in advance, either to some friend, or to the professor whose clinics it is desired to enter, in order to have a place reserved. This is easily done, and frequently saves considerable delay and disappointment.

Taken altogether, there is probably no place in Europe that offers equal facilities for that class of students and practitioners who, having a few weeks or months to spare, wish to take a hasty review of any special subject. All of the short special courses are, however, almost necessarily in a measure incomplete, superficial, and in proportion to the time occupied by them, very expensive.

The more permanent class of students, who settle down for one, two, or three years' steady work, find it much more advantageous to spend at most but a short time here, remaining for the most part either in Berlin, Breslau, Wurtzburg, or even any of the smaller universities, where the courses of study, extending throughout the semester, or the year, are, in most departments at least, much more thorough and complete.

There is one department—that of pathology—in which, in consideration of the amount of material at command, Vienna should far excel all her rivals. The hospital furnishes in the neighborhood of fifteen post-mortems a day, or more than twice the amount which either Virchow, at Berlin, or Waldeger, at Breslau, has at command, and yet either of these places are generally acknowledged to furnish superior advantages for study in this department to anything offered here. In fact, this vast amount of valuable pathological material is here almost literally wasted, as far as any practical use for purposes of instruction are concerned. However well Prof. Rokitansky may deserve the high reputation which he has achieved, he certainly has done but little of late to sustain it.

The diagnosis of syphilis by the microscopic examination of the blood—the supposed great discovery of Dr. Lostorfer—some comments in regard to which appeared in The Examiner for February 15th, is still by no means a generally acknowledged fact, even here. Several of the leading microscopists here, however, are at work investigating the subject. Among others, Prof. Stricker claims to be able to distinguish the characteristic sporules; a few, however, who have not been so fortunate in their investigations, still hold to their unbelief. If this discovery had been announced in Paris, instead of Vienna, we should long ere this have had begun one of those interminable discussions for which the Paris Academy of Medicine are so famous. The old scenes of the unity-duality and the vaccinal-syphilis discussions would have been enacted over again, and, losing sight entirely of all further investigation, the partisans of either side would have arrayed themselves for the grand war of words with a malicious, vindictive energy such as only the excitable Frenchmen are capable of. The German temperament is, however, different. They prefer rather to work much and say little, either party pursuing quietly their investigations, and content to let the truth or falsity of their theories be decided by the future results of their work.

The method of investigation at present pursued by those who are following out observations on the subject, is briefly as follows: A few drops of the blood which it is desired to examine, is placed upon a slide, and being kept constantly moist, is either allowed to stand for two or three days, or the process of decomposition and the consequent formation of sporules can be hastened by keeping the slide for a few hours on a warming stage. By keeping the slide thus slightly warm, as well as moist, the desired growths will sometimes make their appearance within an hour after the process is commenced. These sporules make their appearance quite as abundantly in the healthy as in the syphilitic blood, and it is merely claimed, by the advocates of the discovery, that they are able to distinguish, from careful comparisons made between them, the growths which appear in the specific from those which are found in the healthy blood. F. H. D.

